# Anti-inflammatory Treatment of Kawasaki Disease: Comparison of Current Guidelines and Perspectives

**DOI:** 10.3389/fmed.2021.738850

**Published:** 2021-11-30

**Authors:** Piotr Buda, Joanna Friedman-Gruszczyńska, Janusz Książyk

**Affiliations:** ^1^Department of Pediatrics, Nutrition and Metabolic Diseases, The Children's Memorial Health Institute, Warsaw, Poland; ^2^Department of Pediatric and Congenital Heart Surgery, The Children's Memorial Health Institute, Warsaw, Poland

**Keywords:** Kawasaki disease, vasculitis, guidelines, anti-inflammatory treatment, coronary artery aneurysm, SARS-CoV-2, PIMS-TS, MIS-C

## Abstract

Kawasaki disease (KD), an acute, generalized vasculitis, is associated with an increased risk of coronary heart disease and is the most common cause of acquired heart disease in childhood. The incidence of KD is increasing worldwide. There are numerous international treatment guidelines. Our study aims to perform the first one so far comparison of them. While the gold standard therapy remains still the same (intravenous immunoglobulins and aspirin), there is currently a lack of evidence for choosing optimal treatment for high-risk patients and refractory KD. In this review, we also discuss the treatment of complications of KD and Kawasaki-like phenotypes, present an anti-inflammatory treatment in the light of new scientific data, and present novel potential therapeutic targets for KD.

## Introduction

Kawasaki disease (KD) is an acute, systemic, vasculitis, most commonly occurring in children under 5 years of age. KD, firstly described in Japan in 1967 is the present most common cause of acquired heart disease in childhood (1, 2). The incidence ranges from 138–322/100,000 in Asia, to 4.5–25/100,000 in Europe and the United States of America (3–6). In Great Britain, the number of new cases has doubled in recent years and is now 8.1/100,000 (7, 8). The immunopathogenic mechanism for KD is not completely understood. The epidemiological observations suggest that in genetically predisposed children an environmental agent causes an abnormal hyperactivation of the immune system which results in damage of vascular endothelial cells and systemic vasculitis (8). Many genes responsible for susceptibility to KD have been identified through genome-wide association studies, however they differ within populations (9–15).

The diagnosis of KD should be considered in any child with a febrile exanthematous illness and presence of inflammation, particularly if it persists longer than 4–5 days. The diagnosis of KD is based on clinical criteria, established by the Japanese Ministry of Health Research Committee and adopted by the American Heart Association (16–18). Classic KD is defined as the presence of fever of ≥5 days plus at least ≥4 of the following diagnostic criteria: oral mucosal changes, non-suppurative conjunctival injection, polymorphous skin rash, peripheral changes, including erythema and/or edema of hands and feet, and cervical lymphadenopathy. The incidence of atypical form is increasing. It is more common in infants younger than 6–12 months, the only clinical sign could be fever and abnormalities in laboratory tests, which can cause diagnostic errors. Currently, the diagnosis of KD is only based on clinical and laboratory criteria, interestingly, Wright V et al. found that molecular patterns could enable earlier diagnosis and treatment of KD and reduce inappropriate treatment in those with other diagnoses (19). Although the acute febrile and exanthematous illness may be self-limiting, some patients develop serious complications that are associated with an increased risk of coronary heart disease. The main complication of the disease are coronary artery abnormalities (CAL), however extra coronary complications can occur. The coronary artery aneurysms occur in around 20–30% of untreated cases (19, 20). Coronary artery events (thrombosis, stenosis, intervention, myocardial infarction, death) occurrs in 1–48% of patients with CAL, the incidence depends on the aneurysm *Z* score <10 and on the absolute dimension (21). Up to 4% of cases of untreated KD with CAL will progress to sudden death during the acute phase of the illness as a result of aneurysmal thrombosis formation, myocardial infarction or dysrhythmia (22). In properly treated patients, the risk of permanent changes in coronary arteries decreases significantly (4%) (20, 23, 24). Patients without coronary artery abnormalities have no symptoms or events during follow-up. Medium to long term prognosis after Kawasaki disease is excellent (25). Recurrence of KD has been previously described. It varies between 0.8% in the united states of America to 3% in Japan (5, 26). The proportion of patients suffering from a recurrence increases with age, majority of recurrence occurs within 2 years of the initial presentation (26). In rare cases (0.2%), patient can suffer multiple recurrences (26).

The preliminary understanding of immunogenetic influences the disease susceptibility has already led to treatment with various regimens. The main goal of therapy is to reduce systemic inflammation as early as possible to prevent coronary artery damage.

There are many diagnostic and therapeutic strategies, the aim of this paper is to compare current guidelines and to discuss anti-inflammatory treatment of KD, complications of KD, Kawasaki-like phenotypes and to discuss new potential targets based on new scientific data.

## Treatment Guidelines

There are differences in the scope of the procedure, depending on the recommendations of individual countries.

Most of them are listed below:

– (2014) Guidelines for medical treatment of acute Kawasaki disease: report of the Research Committee of the Japanese Society of Pediatric Cardiology and Cardiac Surgery (2012 revised version) (17).
– (2017) Scientific Statement, which serves as an update to the 2004 American Heart Association guidelines for the diagnosis, treatment, and long-term management of Kawasaki disease (16).
– (2018) European consensus-based recommendations for the diagnosis and treatment of Kawasaki disease—the Single Hub and Access point for pediatric Rheumatology in Europe (SHARE) initiative (27).
– (2021) Revised recommendations of the Italian Society of Pediatrics about the general management of Kawasaki disease (28).

The comparison of various treatment regimens is shown in Tables 1, 2.

**Table 1:** Comparison of guidelines for the treatment of Kawasaki disease.

	**AHA 2017**	**SHARE 2018**	**ISP 2021**	**JPS 2014**
IVIG	High-dose IVIG (2 g/kg given as a single infusion) within 10 days of illness onset but as soon as possible after diagnosis	IVIG (2 g/kg given as a single infusion)	IVIG (2 g/kg), preferably given within the 10th day, better if within the 7th day of illness, but as soon as possible after diagnosis	IVIG—single use (2 g/kg per day) orIVIG—modified single use (1 g/kg per day for 1 or 2 days continuously) or IVIG –divided dosing (200–400 mg/kg per day, over 3–5 days)
IVIG resistance—definition	Persistent or recrudescent fever at least 36 hours and <7 days after completion of first IVIG infusion	Ongoing fever and/or persistent inflammation or clinical signs ≥ 48 h after receiving IVIG as a single dose of 2 g/kg.Laboratory values that can be important in assessing risk stratification for IVIG resistance: low sodium, raised bilirubin, raised ALT, low platelet count, high CRP, low albumin	Failure in the response to IVIG—recrudescent fever reoccurring or persisting 36–48 h after IVIG infusion	Persistent fever after 48 h of starting IVIG
High-risk patients—definition	Not defined criterias for high-risk children outside Japan. In Japan patients at high risk for non-response to IVIG are defined by scoring systems (Kobayashi, Sano)	Patients with severe KD: IVIG-resistant (see above), Kobayashi score ≥5, features of HLH, shock, children under the age of 1 year, children with coronary and/or peripheral aneurysms	Children <12 months or those having CRP higher than 200 mg/l, severe anemia at disease onset, albumin level below 2.5 g/dl, liver disease, overt coronary artery aneurysms, macrophage activation syndrome or septic shock	According to representative scoring systems for evaluating potential IVIG resistance (Kobayashi, Egami, Sano)
ASA moderate-high dose	Administration of moderate (30–50 mg/kg) to high-dose (80–100 mg/kg) ASA is reasonable until the patient is afebrile, although there is no evidence that it reduces coronary artery aneurysms. There are no data to suggest that either dose of ASA is superior	All patients diagnosed with KD who are treated with IVIG should be treated with aspirin at a dose of 30–50 mg/kg/day until fever has settled for 48 h, clinical features are improving, and CRP levels are falling	Treatment of KD is completed by ASA given at a daily dosage of 30–50 mg/kg in the acute phase of KD until 48 h after the disappearance of fever, then switched to the anti-platelet dose (3–5 mg/kg once daily). When GCS are given in patients classified as high risk, ASA is given in low dose (3–5 mg/kg)	Febrile period: oral dose of 30–50 mg/kg/day, in 3 divided doses
ASA low dose	Reducing the ASA dose after the child has been afebrile for 48–72 h. Other clinicians continue high-dose ASA until the 14th day of illness and at least 48–72 h after cessation of fever	The dose of aspirin should subsequently be reduced to an antiplatelet dose of 3–5 mg/kg once daily when fever and inflammation have subsided	Low-dose ASA must be continued until 6–8 weeks in children without CAL and continued in children with CAL until the resolution of coronary artery lesions	48–72 h after defervescence, dosage can be reduced to one dose of 3–5 mg/kg per day

*ALT, alanine aminotransferase; ASA, aspirin; CAL, coronary artery abnormalities; CRP, C-reactive protein; HLH, hemophagocytic lymphohistiocytosis; IVIG, intravenous immunoglobulins; KD, Kawasaki disease; AHA, American Heart Association; SHARE, The European Single Hub and Access point for pediatric Rheumatology in Europe; ISP, Italian Society of Pediatrics; JPS –Japan Pediatric Society*.

**Table 2:** Treatment options for IVIG-resistant KD patients and refractory KD.

	**AHA 2017**	**SHARE 2018**	**ISP 2021**	**JPS 2014**
IVIG resistance—treatment	IVIG or IVIG + GCS or Infliximab It is reasonable to administer a second dose of IVIG (2 g/kg) to patients with persistent or recrudescent fever at least 36 h after the end of the first IVIG infusion	GCS +/– IVIGA second dose of IVIG is at the discretion of the treating physician	IVIG or IVIG + GCS In non-responder patients with KD treatment requires a second infusion of IVIG and—in case of failure—pulses of methylprednisolone (30 mg/kg/day) for 3 consecutive days, followed by oral prednisone (2 mg/kg/day, then gradually tapered)	IVIG + GCSIVIG in combination with either prednisolone or methyloprednisolone
High-risk patients—first line treatment	IVIG + ASA +/− GCS. Administration of a longer course of corticosteroids (e.g., tapering over 2–3 weeks), together with IVIG 2 g/kg and ASA, may be considered for treatment of high-risk patients with acute KD, when such high risk can be identified in patients before initiation of treatment	IVIG + GCS + ASACorticosteroid treatment should be given to patients with severe KD.Treatment should not be delayed while awaiting echocardiography.Two regimens would be reasonable (see below)	IVIG + GCS + ASA In high-risk patients with KD initial treatment should include: IVIG + single intravenous pulse of methylprednisolone (30 mg/kg/day) + low-dose aspirin (3–5 mg/kg/day). In case of failure treatment should be implemented with a further infusion of IVIG and three pulses of intravenous methylprednisolone (30 mg/kg/day, followed by prednisone: 2 mg/kg/day, then gradually tapered) + low-dose aspirin (3–5 mg/kg/day)	IVIG + GCS + ASASuch patients should be treated with 2 g/kg of IVIG in combination with either 2 mg/kg per day prednisolone or 30 mg/kg per day intravenous methylprednisolone pulseIf the patients fail to respond to these treatments, a third-line treatment will be upgraded to a second-line treatment
GCS	Single-dose pulse methylprednisolone should not be administered with IVIG as routine primary therapy for patients with KD. Administration of a longer course of corticosteroids (e.g., tapering over 2–3 weeks), together with IVIG 2 g/kg and ASA, may be considered for treatment of high-risk patients with acute KD, when such high risk can be identified in patients before initiation of treatment. Administration of high-dose pulse steroids (usually methylprednisolone 20–30 mg/kg intravenously for 3 days, with or without a subsequent course and taper of oral prednisone) may be considered as an alternative to a second infusion of IVIG or for retreatment of patients with KD who have had recurrent or recrudescent fever after additional IVIG. Administration of a longer (e.g., 2–3 weeks) tapering course of prednisolone or prednisone, together with IVIG 2 g/kg and ASA, may be considered in the retreatment of patients with KD who have had recurrent or recrudescent fever after initial IVIG treatment	Corticosteroid treatment should be given to patients with severe KD (see high-risk patients, Table 1)	In high risk patients. In case of failure treatment	In patients suspected of being IVIG resistant on the basis of clinical symptoms and laboratory findings.In patients found to be IVIG resistant after first-line IVIG treatment
Treatment options for IVIG-Resistant KD patients and Refractory KD	IVIG GCS Infliximab CsA ANA CYP PE — CsA: Administration of cyclosporine may be considered in patients with refractory KD in whom a second IVIG infusion, infliximab, or a course of steroids has failed. Administration of immunomodulatory monoclonal antibody therapy (except TNF-α blockers), cytotoxic agents, or (rarely) plasma exchange may be considered in highly refractory patients who have failed to respond to a second infusion of IVIG, an extended course of steroids, or infliximab	IVIGGCSInfliximab—TNF-alpha blockade (e.g., infliximab) should be considered in KD patients with persistent inflammation despite IVIG, aspirin and corticosteroid treatment, after consultation with a specialist unit.The use of DMARDs such as cyclosporin, cyclophosphamide and methotrexate, along with anakinra and plasma exchange, cannot be recommended, except on an individual basis after consultation with a specialist unit	IVIG GCS Infliximab Anakinra Canakinumab — Current evidence supports the use of infliximab as rescue therapy in IVIG- and methylprednisolone-refractory patients with KD; IL-1 blockade with anakinra is highly promising in treating the most dramatically severe multi-refractory patients with KD, with potential benefits also on the cardiovascular complications	IVIGGCSInfliximabUlinastatinCsAMethotrexatePE—
Prednisone/prednisolone	Prednisolone 2 mg/kg i.v. divided every 8 h until afebrile, then prednisone orally until CRP normalized, then taper over 2–3 weeks	–	After intravenours methylprednisolone treatment. Prednisone at the initial dose of 2 mg/kg/day, then tapered up to the resolution of symptoms and normalization of CRP	During fever: 2 mg/kg/day of prednisolone, i.v. in 3 divided dosesAfter defervescence: Once patient is no longer febrile and general status has improved, prednisolone is given orally. When CRP normalizes, the dose of prednisolone is tapered over 15 days, in 5 day steps, from 2 mg/kg/day in 3 divided doses to 1 mg/kg/day in 2 divided doses to 0.5 mg/kg/day in a single dose
Methylprednisolone	Usually 20–30 mg/kg intravenously for 3 days, with or without a subsequent course and taper of oral prednisone	Regimen 1: methylprednisolone 2 ×0.8 mg/kg for 5–7 days or until CRP normalizes; then convert to oral prednisone/prednisolone 2 mg/kg/day and wean off over next 2–3 weeks.Regimen 2: methylprednisolone 10–30 mg/kg (up to maximum of 1 g/day) once daily for 3 days followed by oral prednisone/prednisolone 2 mg/kg per day until day 7 or until CRP normalizes; then wean over next 2–3 weeks	In high-risk patients with KD initial treatment should include: IVIG + single intravenous pulse of methylprednisolone (30 mg/kg/day) + low-dose aspirin (3–5 mg/kg/day). In case of failure treatment should be implemented with a further infusion of IVIG and three pulses of intravenous methylprednisolone (30 mg/kg/day, followed by prednisone: 2 mg/kg/day, then gradually tapered) + low-dose aspirin (3–5 mg/kg/day). In low-risk KD patients resistant to two previous infusions of IVIG: pulses of methylprednisolone (30 mg/kg/day) for 3 days. followed by oral prednisone (2 mg/kg/day, then gradually tapered)	When used in combination with first-line IVIG: 1 dose of 30 mg/kg methylprednisolone. When used to treat IVIG-resistant patients: 30 mg/kg methylprednisolone once a day, for 1–3 days. Some reports suggest additional prednisolone (started at 1–2 mg/kg/day and gradually tapered over a period of 1–3 weeks) after methylprednisolone
Infliximab	Administration of infliximab (5 mg/kg) may be considered as an alternative to a second infusion of IVIG or corticosteroids for IVIG-resistant patients. Single infusion: 5 mg/kg IV given over 2 h	Infliximab should be considered in KD patients with persistent inflammation despite IVIG, aspirin and corticosteroid treatment, after consultation with a specialist unit	Current evidence supports the use of infliximab, a chimeric monoclonal antibody against TNF-α, as rescue therapy at a single intravenous dose of 5 mg/kg of body weight (given in 2 h) for IVIG- and corticosteroid resistant KD patients	i.v. drip infusion of 5 mg/kg (may only be given once)
Anakinra	2–6 mg/kg given by subcutaneous injection	The use of DMARDs such as ciclosporin, cyclophosphamide and methotrexate, along with anakinra and plasma exchange, cannot be recommended, except on an individual basis after consultation with a specialist unit	In children with a refractory KD, given subcutaneously at a daily dose of 4–8 mg/kg of body weight for an overall period of 15 days or for a longer period, depending on the specific clinical scenery	–
Cyclosporin A	i.v.: 3 mg/kg divided every 12 h p.o.: 4–8 mg/kg divided every 12 h. Adjust dose to achieve trough 50–150 ng/mL; 2-h peak level 300–600 ng/mL	The use of DMARDs such as cyclosporin, cyclophosphamide and methotrexate, along with anakinra and plasma exchange, cannot be recommended, except on an individual basis after consultation with a specialist unit	4 mg/kg/day in 2 doses p.o.; in case of persistence of fever the dosage can be increased to 5–8 mg/kg/day; administered until CRP normalization or for 10–14 days	Start on 2 divided oral doses (1 each before meal) of 4–5 mg/kg/dayTarget trough level: 60–200 ng/mL
Plasma Exchange	Plasma exchange should be reserved for patients in whom all reasonable medical therapies have failed	The use of DMARDs such as cyclosporin, cyclophosphamide and methotrexate, along with anakinra and plasma exchange, cannot be recommended, except on an individual basis after consultation with a specialist unit	–	Displacing solution set at 5% albumin; 1–1.5 × the patient's circulating plasma volume is exchanged. Usually given for 3 continuous days (upper limit: 6 days)

*ANA, anakinra; ASA, aspirin; CsA, cyclosporin A; CRP, C-reactive protein; CYP, cyclophosphamide; DMARDs, disease-modifying antirheumatic drugs; GCS, glucocorticosteroids; INF, inliximab; IVIG, intravenous immunoglobulins; KD, Kawasaki disease; PE, plasma exchange; AHA, American Heart Association; SHARE, The European Single Hub and Access point for pediatric Rheumatology in Europe; ISP, Italian Society of Pediatrics; JPS, Japan Pediatric Society*.

There are also guidelines on the long-term management of patients who have vascular complications of KD. This therapy is individualized, it usually consists of medicines for heart conditions (antithrombotic therapy, statins, beta-blockers, interventional cardiology, cardiac surgery), though this topic exceeds the aim of this paper.

– (2020) Japanese Circulation Society Working Group 2020 Guideline on Diagnosis and Management of Cardiovascular Sequelae in Kawasaki Disease (29).
– (2020) Expert consensus statement “Lifetime cardiovascular management of patients with previous Kawasaki disease” (30).

It is also worth to mention that in 2020 Japan Pediatric Society presented the revision of guidelines for Kawasaki disease (6th revised edition) but only in the context of the diagnosis.

– (2020) Japan Pediatric Society: Revision of diagnostic guidelines for Kawasaki disease (6th revised edition) (18).

## Standard Treatment of Kawasaki Disease

All above mentioned management guidelines are consistent with the first-line treatment. Treatment of acute illness with intravenous immunoglobulin (IVIG) and acetylosalic acid (ASA) is now the gold standard recommendation. Differences concerning aspirin dose are presented in Table 1.

### Intravenous Immunoglobulins (IVIG)

Currently, the most effective anti-inflammatory treatment for KD is an early transfusion of intravenous immunoglobulins. Randomized clinical trials performed in the 1980s suggested that IVIG reduced the prevalence of persistent coronary artery lesions (CAL) (21, 31). The systematic review by the Cochrane Collaboration states that CAL development can be reduced by a single dose of 2 g/kg IVIG given before the 10th day after onset, thus, high-dose IVIG is still the first-line treatment of KD according to all current guidelines (Table 1) (24).

The molecular mechanisms of IVIG for anti-inflammation in KD remain unclear. Potential mechanisms include the blockade of the Fc receptor, neutralization of the pathogenic products of unknown infectious agents, immune-modulatory effects, stimulation of suppressor activity, and modulation of the cytokines (9, 14, 32–34). Multiple studies show that ~10–28% of patients are resistant to first-line treatment (no resolution of fever, recurrent fever, no / slight decrease in inflammation parameters) (20, 34). The definition of IVIG resistance varies according to different recommendations (Table 1). Many studies have been conducted to identify predictive factors of resistance to IVIG therapy. Xuan Li et al. performed a meta-analysis of 4,442 children with KD and identified the clinical features and laboratory factors: the initial administration of IVIG ≤ 4.0 days after the onset of symptoms, increased erythrocyte sedimentation rate (ESR) and decreased hemoglobin and platelet counts, oral mucosa alterations, cervical lymphadenopathy, swelling of extremities, and polymorphous rash (35). Yan et al. in their systematic review and meta-analysis confirmed that gender, IVIG resistance, IVIG treatment beyond 10 days of onset of symptoms and increased C-reactive protein (CRP) level are all significant risk factors for CAL (36). Zheng et al. performed the first meta-analysis that revealed the strongest association between the incidence of CAL and IVIG resistance (37). There is currently no universally accepted classification system to evaluate KD severity. Many predictive models that were designed to evaluate the possibility of IVIG resistance were proposed (38–47). Scoring systems (Kobayashi, Sano, Egami) most commonly used in clinical practice include following parameters: hyponatremia, prolonged illness duration, elevated C-reactive protein, aspartate transaminase, alanine transaminase (ALT), bilirubin, neutrophil ratio, low count of platelets. The problem is that there are no such predictive instruments or scores outside Japan, the effectiveness of such scores has not been confirmed in large-scale prospective cohort studies or meta-analyses. Kuo et al. used a novel approach by conducting a genomewide association analysis to develop a risk score for IVIG resistance (48). However, it is unknown whether one universal prediction model can be developed for all populations or population-specific prediction models will be required (49). Recently, Piram et al. identified predictors of IVIG resistance and presented a new score with good sensitivity and acceptable specificity in a non-Asian population (50). Predictors of secondary treatment after initial IVIG were hepatomegaly, ALT level ≥30 IU/L, lymphocyte count <2,400/mm3 and time to treatment <5 days. These findings have not yet been used to current guidelines.

The development of CAL despite IVIG treatment ranges from 19 to 42% (51, 52). A genetic contribution to CAL is likely as before effective therapy with IVIG was introduced, only 25–30% of affected children developed CAL (22, 53). Many genes and chromosomal regions have been identified through genome-wide association studies to have an association with KD and CAL formation (10, 14, 53, 54). Genes responsible for susceptibility and CAL formation may be different between populations. The neutrophil antigen 1 allotype in the extracellular domain 1 of *Fc*γ*R3B* has been identified as a major risk factor for IVIG refractoriness and persistent CAL (32). In the future, risk scores may include genetic testing for high-risk small nucleotide polymorphisms (SNPs).

### Acetylsalicylid Acid (ASA)

Aspirin (ASA, aspirin) inhibits platelet function through irreversible inhibition of cyclooxygenase (COX) activity and blocks the synthesis of prostaglandins. The mechanism of action of aspirin depends on dosage, medium-high doses are usually given to obtain the anti-inflammatory effect, low doses inhibit platelet aggregation. ASA has been used in the treatment of KD for many years and is approved for all patients with KD. High-dose (80–100 mg/kg) and medium dose (30–50 mg/kg) acetylsalicylic acid have been recommended as standard treatment during the acute febrile phase by the American Heart Association and Japanese Society of Pediatric Cardiology and Cardiac Surgery, respectively (16, 18). The optimal dose of ASA remains controversial, however. Although high-dose aspirin shortens fever duration, researchers of many recent studies found that the use of medium- or higher-dose ASA in acute Kawasaki disease did not prevent CAL (54–58). Considering the risk of drug toxicity and the lack of evidence for prevention of CALs, the role of aspirin in the acute phase of KD needs to be reassessed and a future randomized controlled trial is needed to determine the optimum dose of ASA. Clinical trials comparing the efficacy of IVIG alone and IVIG plus high-dose aspirin in KD are ongoing. The duration of high-dose ASA administration varies across institutions. Some physicians recommend conversion to an antiplatelet dose of ASA after the child has been afebrile for 48–72 h. Others continue high-dose ASA until the 14th day of illness. Low-dose ASA is continued until the patient has no evidence of CAL by 6–8 weeks after onset of fever. For children who develop CAL, ASA may be continued indefinitely (16).

It is unclear what dose (anti-inflammatory vs. anti-platelet) of aspirin should be used with simultaneous supply of glucocorticosteroids (GCS) and whether to give aspirin at all (since GCS are anti-inflammatory and the combined use of both drugs increases their side effects).

Interestingly, only Italian guidelines indicate that patients treated with GCS as a first-line treatment need to be treated simultaneously with low dose ASA instead of high-dose ASA. Such strategy is reasonable but some authors concluded that in the absence of comparative studies, it is practiced to use both drugs.

## Second-Line Treatment

Patients who are at increased risk of CAL, unresponsive to IVIG may be treated with second dose of IVIG, glucocorticosteroids, infliximab or other immunosuppressive agents. To date, there have been no robust clinical trials comparing second-line treatment options for IVIG resistant KD. Treatment choice varies according to different recommendations (Tables 1, 2).

### Glucocorticosteroids (GCS)

GCS inhibit the transcription of most pro-inflammatory cytokines *(*IL*-*1, IL*-*2, IL*-*6, IL*-*8, interferon-γ, and tumor necrosis factor-α) (59). They also inhibit the proliferation of T and B lymphocytes, Langerhans cells, decrease adhesive molecule expression. Because of their effects on a broad range of innate and adaptive responses and effect on multiple types of immune cells, GCS are remarkable helpful in managing many of autoinflammatory and autoimmune diseases (60, 61). Corticosteroids are usually administered in all vasculitides due to their anti-inflammatory effect, but the use of GCS in children with KD is still controversial and varies depending on individual recommendations (16, 27, 28). In 2007, a multi-center prospective randomized, placebo-controlled, double-blinded study found no significant difference in coronary z scores or in the duration of fever in those treated with corticosteroids in addition to IVIG (62). Subsequent Japanese studies have shown that the addition of corticosteroids significantly decreases the risk of CAL; however, these studies included only patients classified as patients with a high risk of IVIG resistance based on Asian risk scores (63–66). In 2016 meta-analyses showed that the frequency of CAL was significantly lower in children that received GCS with IVIG than IVIG therapy only (67). Sixteen comparative studies were analyzed. It is worth noting that most included studies were conducted in Japan. Whether these results are applicable to other countries remains to be elucidated. Others found that long-term steroid treatment should be considered in all children diagnosed with the disease (68). Yang et al. stated that GCS treatment, combined with IVIG, reduces the incidence of coronary aneurysms, but only in Japanese patients, which was not observed in other nations' patients (69). Thus, these studies' conclusions should not be extrapolated to non-Asian populations due to the possible influence of various environmental, genetic, and economic factors on the effects of therapy (70). The current American Heart Association guidelines do not recommend routine use of adjunctive corticosteroids, but rather consideration for high-risk patients. The administration of a longer course of corticosteroids together with IVIG and ASA may be considered for treatment of high-risk patients, when they can be classified before initiation of treatment. Administration of high-dose pulse steroids may be considered as an alternative to the second infusion of IVIG or for retreatment of patients with KD who have had recurrent or recrudescent fever after additional IVIG (16). According to the SHARE guidelines, adjunctive primary GCS treatment should be given to children: who are IVIG resistant, have a Kobayashi score ≥4 or developed MAS/HLH and/or shock. The panel of experts defined additional ‘high-risk groups’ who might benefit from primary adjunctive GCS: infants <1 year of age and children presented with coronary and/or peripheral aneurysms at diagnosis. It is unclear whether corticosteroids should be used in children with less severe KD, and the optimal corticosteroid dosing regimen to use is uncertain. Italian and Japanese guidelines indicate the use of GCS for patients suspected of being IVIG resistant on the basis of clinical symptoms and laboratory findings and for patients found to be IVIG resistant after first-line IVIG (18, 28). The problem is that there are no predictive instruments or scores for reliable identification of high-risk children outside Japan, further research is needed to test the efficacy of GCS in this population. KD-CAAP is a multi-center, randomized trial comparing the effectiveness of corticosteroids with standard treatment vs. standard treatment alone to prevent KD heart complications. The study is ongoing.

### Infliximab

Monoclonal antibodies may target the presumed key-cytokines involved in KD pathogenesis, particularly tumor necrosis factor (TNF)-α and interleukin (IL)-1 (71, 72). Elevated serum TNF-alpha is elevated in patients with KD and it correlates with the development of CAL. Infliximab is a chimeric murine/human IgG1 monoclonal antibody that binds specifically to TNF-alpha with high affinity and neutralizes the biological activity of soluble TNF-α (73). Among monoclonal antibodies, infliximab is the most widely tested drug in KD. It is safe and well-tolerated drug that reduces fever duration and inflammation, but the addition of infliximab to primary treatment in acute Kawasaki disease did not reduce treatment resistance. No trials have evaluated its use as adjunctive therapy in patients with early evidence of CAL (74). Thus, current guidelines supports the use of infliximab, as a rescue therapy at a single intravenous dose (5 mg/kg of body weight given in 2 h) for IVIG- and corticosteroid resistant KD patients.

The efficacy of another tumor necrosis factor-α receptor blocker (etanercept) was also evaluated (75, 76). However, the disadvantage of etanercept is that it only binds to circulating and not cell-bound TNF-alpha which could potentially impair its efficacy (77).

### Anakinra

The IL-1 signaling pathway seems to be key to the pathogenesis of KD, especially in the development of coronary artery aneurysms (78). Upregulated IL-1 pathway genes and elevated IL-1 concentrations have been demonstrated in the peripheral blood of KD patients during the acute phase of the disease (79, 80). Weng et al. showed that polymorphisms in the genes coding for IL-1 (-31 CC and−511 TT) were associated with a greater risk of resistance to IVIG treatment (81). The use of IL-1 inhibitors in patients with KD has been reported, but data are largely limited to small case series. Ferrara et al. summarized the scientific literature related to the use of anakinra, analyzing preclinical and clinical data (82). Reasons for using anakinra are as followed: Kawasaki disease shock syndrome, macrophage activation syndrome, persistent fever and laboratory abnormalities, worsening of coronary aneurysms, coronary aneurysms and increased proBNP levels. The dose ranged from 1 to 10 mg/kg/day; the duration ranged from 6 days to 6 months (83–88). According to compared recommendations only IPS mentioned about the duration of treatment for an overall period of 15 days or for a longer period, depending on the specific clinical scenery (28, 89). In the largest study concerning anakinra (KAWAKINRA), starting doses were 2 mg/kg of anakinra (4 mg/kg in patients who were age <8 months and who weighed ≥5 kg), and the dose was increased up to 6 mg/kg every 24 h if the patient's was febrile. Treatment duration was 2 weeks. Almost all patients (sixteen patients included) received a clinical benefit (reducing fever, markers of systemic inflammation, and coronary artery dilatation), and no relevant side effects were noted. Authors concluded that anakinra may be considered as an option after the failure of the first IVIG infusion, especially in patients with coronary involvement (90). Mastrolia MV et al. have recently reported two cases of children, diagnosed with KD, non-responsive to two doses of intravenous immunoglobulins, successfully treated with ANA, without prior use of steroids (91). Further studies are planned/ongoing to reveal its clinical significance (ANACOMP, ANAKID) and to better define the place of IL-1 blockade in KD step-up treatment.

Interestingly, other anti-IL drugs could be regarded as an alternative treatment. Canakinumab is a human monoclonal antibody targeted at IL-1β, with no cross-reactivity with other members of the IL-1 family. It has been authorized for the treatment of systemic juvenile idiopathic arthritis and different hereditary autoinflammatory syndromes. According to ISP guidelines using a single subcutaneous injection of 4 mg/kg/dose of canakinumab may be also a future option for cases of IVIG-resistant and corticosteroid-resistant KD (28).

### Cyclosporin A

Cyclosporin A is a calcineurin inhibitor that exerts its immunosuppressive effects through the down-regulation of NFAT (nuclear factor of activated T cells) transcription factor, and suppresses cytokine production such as IL-2 by inhibiting nuclear factor of activated T cells (17, 92). It has been studied as both a second-line therapy and as rescue therapy for KD.

The largest study (KAICA trial) was conducted on Japanese participants. Hamada et al. found that combined primary therapy with IVIG and cyclosporin was safe and effective for favorable coronary artery outcomes in Kawasaki disease patients who were predicted to be unresponsive to IVIG (93). Despite this CsA is reserved only for refractory KD according to current guidelines (including Japanese) (16, 17, 27, 28).

### Other Treatment

Cyclophosphamide, methotrexate, ulinastatin have also been used in refractory-KD however according to all current guidelines these medicaments should only be considered in severe refractory cases because of potential adverse reactions and better experience with previously mentioned medicaments (77, 94–97). Plasma exchange (PE) could act via mechanical removal of inflammatory cytokines and was used in patients with refractory KD (17, 98, 99). The largest series reported to date included 125 patients who were resistant to IVIG and treated with plasma exchange (100). Authors conclude that outcomes of PE for Kawasaki disease refractory to IVIG are favorable, although not statistically significant. Because PE is a high-risk procedure and there are no controlled clinical trials it could be considered only in extreme cases of refractory KD.

## Treatment of Other Clinical Conditions Related to KD

### Macrophage Activation Syndrome (MAS)

Macrophage Activation Syndrome (MAS) is a form of secondary hemophagocytic lymphohistiocytosis (HLH). It is a life-threatening systemic extreme-inflammatory syndrome caused by multifactorial immune dysregulation and pathological hyperactivation of the immune system. The most common form of HLH is MAS in the course of systemic-onset juvenile idiopathic arthritis (so-JIA) but it could also occur as the manifestation of Kawasaki disease (101, 102). Macrophage activation syndrome is characterized by fever, hepato- and/or splenomegaly, non-characteristic skin lesions, lymphadenopathy, coagulopathy, central nervous system dysfunction. Symptoms of the respiratory system and heart failure could also be present. Uncharacteristic clinical symptoms often mistakenly suggest sepsis, are accompanied by more characteristic additional diagnostic work-up. Cytopenias, hypofibrinogenemia, hypertriglyceridemia, hyperferritinemia are the most common findings. MAS may be frequently under-recognized in children with KD because there are no distinct criteria for MAS complicating KD (103). Some authors recommend that Histiocyte Society criteria may be used for the diagnosis of MAS in KD (104, 105). The MAS criteria are validated for systemic juvenile idiopathic arthritis, but they are commonly used by other physicians for other systemic autoinflammatory diseases such as Kawasaki disease (106, 107). KD patients with MAS show high intravenous immunoglobulin (IVIG) resistance and coronary complications, they usually present with hepatosplenomegaly, cytopenia, liver dysfunction, hyperferritinemia, elevated serum LDH, hypofibrinogenemia, hypertriglyceridemia (103, 104).

The main goal of the therapy of MAS is to stop “cytokine storm,” the treatment should be implemented as soon as possible. The antimicrobial therapy usually is necessary because of fact that each form of HLH is triggered by an infectious agent. The chemotherapy protocol (HLH-2004) including etoposide, cyclosporine, dexamethasone, and transplantation of hematopoietic stem cells is widely used in primary HLH. For patients with acquired HLH there are no recommendations and guidelines. Glucocorticosteroids, intravenous immunoglobulins and cyclosporine A are commonly used. Anti-cytokines antibodies, cyclophosphamide, vincristine, anti-thymocyte globulin, granulocyte-colony stimulating factor, plasma exchange or hemofiltration could be used in severe and refractory HLH (102, 108–110). Some authors start with HLH-2004 protocol for secondary HLH (102, 105). Inappropriate treatment such as immunosuppression monotherapy and a delay in the start of treatment may be one of the main unfavorable prognostic factors in patients with MAS. The combined immunosuppression (high-dose GCS in combination with CsA and IVIG) is usually given as the initial therapy for patients with secondary HLH (102, 108, 109, 111). The commonly used treatment in children with MAS and KD is combination therapy with GCS, IVIG, cyclosporine, IL-1 blockers (103, 104, 112). Furthermore, in MAS there is a high risk of thrombosis because of the massive activation of the coagulation cascade. In cases of highly elevated level of D-dimers (seen especially in MAS and other hyperinflammatory conditions like pediatric inflammatory multisystem syndrome-temporally associated with SARS-CoV-2) the use of anticoagulant drugs (e.g., enoxaparin) could be required.

Appropriate treatment of MAS requires the collaboration of pediatric, infectious disease, and intensive care unit specialists with other experts such as rheumatologists, immunologists, hematologists.

### Pediatric Inflammatory Multisystem Syndrome-Temporally Associated With SARS-CoV-2 (PIMS-TS)

Since late April 2020, many articles have been published describing the increasing incidence of Kawasaki-like disease after the beginning of the SARS-CoV-2 epidemic (107, 113–117). The new entity was proposed so-called Pediatric Inflammatory Multisystem Syndrome-temporally associated with SARS-CoV-2 (PIMS-TS). Multisystem inflammatory syndrome in children (MIS-C) is an alternative name proposed in the United States of America (USA) and adopted by the World Health Organization (WHO). Whether this is a particular form of KD triggered by SARS-CoV-2 or a different entity is still a matter of debate. Some of the clinical manifestations of PIMS-TS mimic KD and MAS. Children with PIMS-TS are usually older at disease onset, classic mucocutaneous symptoms are less common, gastrointestinal and respiratory symptoms are more frequently observed. Patients are at higher risk to develop myocarditis with heart insufficiency and require longer time in the hospital and ICU admittance, for the occurrence of shock, need of vasoactive agents, and invasive ventilation. Many treatment protocols recommends the use of IVIG and aspirin with/without high-dose corticosteroids as first-line therapy. Indications for the use of GCS and dosing depends on the phenotype of the disease and differs in many medical centers. Approximately 30–80% of patients do not respond to IVIG alone and may require adjunctive immunomodulatory therapy to control inflammation. This is in contrast to classic KD where IVIG resistance has been seen in <15% of patients. Anakinra is the most common anticytokine drug used in a subgroup of children with PIMS-TS in many medical institutions, given in cases of persistent severe inflammatory state despite previous treatment (113, 116, 118–121). Treatment with tocilizumab (humanized anti-IL-6 receptor antibody, inhibiting IL-6) or infliximab was also initiated in patients with PIMS-TS with a favorable outcomes. The effect of immunomodulatory therapy needs further evaluation in both observational and trial settings to determine the influence on inflammation (116, 118, 122).

## Perspectives

### KD and SoJIA

Systemic-onset juvenile idiopathic arthritis (so-JIA) is a systemic inflammatory disease classified as a subtype of juvenile idiopathic arthritis. It is associated with dysregulation of the innate immune system, suggesting that it belongs to the spectrum of autoinflammatory disorders. KD and so-JIA share many common clinical and laboratory features. So-JIA can be initially diagnosed as KD and vice versa (123–125). CAL can be also found in soJIA. Most children with soJIA and coronary artery dilatations are classified initially as KD and treated with multiple doses of IVIG. Although KD and so-JIA could mimic each other at the presentation, the follow-up is quite different. Non-responsiveness to standard therapy with GCS and classical disease-modifying antirheumatic drugs is not uncommon in children with so-JIA. Recently, biologic agents that specifically inhibit the cytokines interleukin (IL)-1 and IL-6 have demonstrated remarkable clinical effectiveness and confirmed the importance of these cytokines in the process of so-JIA (126). The three IL-1 blockers that have been tested so far (anakinra, canakinumab, and rilonacept) have all been proven effective and safe, although only canakinumab is currently approved for use in so-JIA (127–130). IL-18 is another proinflammatory cytokine elevated in so-JIA and may represent a pathogenic link between so-JIA and MAS (131). Based on this, some authors suggested using exogenous IL-18BP (IL-18 binding protein) as a novel therapeutic approach for inflammatory diseases (132). A recent Phase II trial of recombinant IL-18BP (tadekinig alfa) showed promising results for adult-onset Still's disease (133). Some authors found that it could be useful in resistant systemic juvenile idiopathic arthritis and recurrent macrophage activation syndrome (134). Interestingly, IL-18 is also elevated in the acute phase of KD and may be protective for those at high-risk for treatment failure (135). Above mentioned findings warrant future research on these drugs as a promising therapeutic option also in Kawasaki disease.

### Potential Therapeutic Target

Many recent studies found novel immunobiological pathways involved in KD and allowed to identify potential therapeutic targets for KD, they are listed in Table 3 (15, 37, 136–147). Literature data indicate that researchers focused especially on JAK / STAT pathway in the context of vasculitis, thus it could be regarded as a most promising potential target.

**Table 3:** Potential therapeutic target for Kawasaki disease.

**Potential target**	**Description**	**References**
S100A12	One of serum protein-based biomarkers of KD (S100A12 promoted *in vitro* neutrophil infiltration which is the cause of *in vivo* CAL formation	(136)
Platelet miR-223 or VSMC PDGFRβ	Uptake of platelets and platelet-derived miRNAs influences vascular smooth muscle cell phenotype *in vivo*	(137)
ANXA1	Annexin A1 (ANXA1) is an endogenous anti-inflammatory agent and pro-resolving mediator involved in inflammation-related diseases	(138)
NLRP3	NLRP3 inflammasome is a large multiprotein complex that plays a key role in IL-1β-driven sterile inflammatory diseases	(139)
ITGAM	In KD coronary artery lesions, Integrin αM (ITGAM) might enhance subacute/chronic vasculitis, resulting in the transition of smooth muscle cells to myofibroblasts and their subsequent proliferation	(140, 141)
JAK/STAT	RPN2 inhibits autophagy via STAT3 (signal transducer and activator of transcription-3) and NF-κB pathways STAT3 is activated by interleukin 6, a pro-inflammatory cytokine that is involved in early innate immune reactivity, and present in the acute phase of KD JAK1/STAT3 signaling pathway is activated in some systemic vasculitides through the activation of Th1/Th17-type cytokines such as IL-2, interferon (IFN-γ), IL-6, IL-17, and IL-23	(15, 37, 142–146)
STING	Over-activation of the STING-pathway (Stimulator of interferon (IFN) genes), could increase the risk of delayed aneurysms in KD and COVID-19 vasculitis	(147)
KCa3.1	KCa3.1 (calcium-activated potassium channel) blockade of macrophages suppresses inflammatory reaction leading to mouse coronary artery endothelial cell injury in a cell model of KD by hampering the activation of NF-κB and STAT3 signaling pathway	(37)

## Conclusions

IVIG and ASA are now the gold standard recommendation for the treatment of Kawasaki disease according to all guidelines. However new scientific data indicate that in the future this regimen can change. Definition of high-risk patients, as well as the indication for additional treatment in these patients, varies depending on the national recommendations. Stratification of patients and optimalization of the second-line therapy is the most urgent issue in Kawasaki disease and the effect of immunomodulatory therapy needs further evaluation in carefully designed observational and trial settings to determine the effect on inflammation. There is currently a lack of evidence for choosing optimal treatment for refractory KD.

The use of glucocorticosteroids in children with KD is still controversial. Monoclonal antibodies are currently regarded as a rescue therapy, althought some data could indicate that anakinra and infliximab may be considered as an option after the failure of the first IVIG infusion. Other medicaments should only be considered in severe refractory cases because of potential adverse reactions. Results of many ongoing studies are awaited and may provide changes in the future management of KD patients.

So-JIA overlaps clinical and immunological presentation with KD and these findings could encourage to perform further studies based on previous results on so-JIA and other autoinflammatory syndromes. Many recently described immunobiological pathways could serve as a promising potential therapeutic target.

## Author Contributions

PB have made a substantial contribution to the concept or design of the article, or the acquisition, analysis, or interpretation of data for the article, and drafted the article. JF-G and JK revised the article critically for important intellectual content and approved the version to be published. All authors reviewed the results and approved the final version of the manuscript.

## Conflict of Interest

The authors declare that the research was conducted in the absence of any commercial or financial relationships that could be construed as a potential conflict of interest.

## Publisher's Note

All claims expressed in this article are solely those of the authors and do not necessarily represent those of their affiliated organizations, or those of the publisher, the editors and the reviewers. Any product that may be evaluated in this article, or claim that may be made by its manufacturer, is not guaranteed or endorsed by the publisher.
